# Alginate-Assisted Lemongrass (*Cymbopogon nardus*) Essential Oil Dispersions for Antifungal Activity

**DOI:** 10.3390/foods10071528

**Published:** 2021-07-02

**Authors:** Martina Cofelice, Giuseppe Cinelli, Francesco Lopez, Tiziana Di Renzo, Raffaele Coppola, Anna Reale

**Affiliations:** 1 Department of Agricultural, Environmental and Food Sciences (DiAAA) and Center for Colloid and Surface Science (CSGI), University of Molise, Via De Sanctis, I-86100 Campobasso, Italy; m.cofelice1@studenti.unimol.it (M.C.); giuseppe.cinelli@gmail.com (G.C.); lopez@unimol.it (F.L.); coppola@unimol.it (R.C.); 2 Institute of Food Sciences, National Research Council (CNR-ISA), Via Roma 64, 83100 Avellino, Italy; tiziana.direnzo@isa.cnr.it

**Keywords:** essential oil, moulds, antimicrobial, nanoformulations, emulsion, alginate, *Penicillium expansum*, *Aspergillus niger*, *Rhyzopus* spp.

## Abstract

The use of natural compounds as food preservatives is becoming increasingly popular as it is perceived positively by consumers. Among these substances, essential oils have attracted great interest owing to their antioxidant and antimicrobial properties. However, several challenges impair the use of essential oils in food products, such as their degradation or loss during food processing and storage, the strong aroma, even at low concentrations, which may negatively affect the sensory characteristics of food. In this context, the development of nanoformulations able to stabilize essential oils may represent a smart solution to this issue. The aim of the study was to evaluate the efficiency of alginate-based nanoformulations enriched with lemongrass (*Cymbopogon nardus*) essential oil (LEO) and Tween 80 against several fungi namely *Penicillium expansus*, *Aspergillus niger* and *Rhizopus* spp. Firstly, the flow behavior of systems at different concentrations of alginate (1%, 2% and 3% *w*/*w*) were studied. Then, emulsion-based nanoformulations at different concentrations of lemongrass essential oil in the range of 0–2% *w*/*w* were stabilized by a fixed amount of Tween 80, characterized and tested for their antifungal activity. Our results showed that the best nanoformulation able to inhibit *Rhizopus* spp., *Penicillium expansum* and *Aspergillus niger*, for at least 10 days, was constituted by 1% alginate/1.5% LEO/1% Tween 80. Hence, the incorporation of essential oil into nanoformulation systems may represent a valid alternative to overcome the disadvantages that limit the commercial application of essential oils.

## 1. Introduction

Essential oils (EOs) can be defined as complex mixtures of hydrophobic and volatile substances extracted by different methods, such as hydro-distillation, steam distillation, hydro-diffusion and solvent extraction from various parts of the aromatic plants such as flowers, seeds, leaves, barks, herbs, fruits, roots, etc. [1]. Essential oils consist of different substances, such as terpenes, terpenoids, phenolic acids and other aromatic and aliphatic compounds, the composition of which depends on the origin of raw material and extraction method [2]. EOs are generally known to exert various beneficial pharmacological effects, such as anti-allergic and anticancer effects, also presenting neuroprotective, anti-inflammatory, immunomodulatory and anticonvulsant properties [3,4]. EOs and their constituents have been used for a long time as flavoring agents in food and are classified as GRAS (Generally Recognized As Safe) [5].

Besides this, different EOs have demonstrated their efficacy in inhibiting bacterial, fungi and virus replication and as potent antioxidants and free radical scavengers [6,7,8]. The antibacterial activity of different plant EOs is well-documented against both Gram-positive (*Bacillus subtilis*, *Staphylococcus aureus*, *Listeria monocytogenes*) and Gram-negative (*Escherichia coli*, *Salmonella typhimurium*, *Pseudomonas aeruginosa*, *Camplyobacter* spp.) bacteria while studies on antifungal and antimycotoxigenic activities of EOs are more limited [9]. Due to their natural antimicrobial and antioxidant effect, which helps to extend the shelf-life of commodities, EOs are widely used in the food industry mainly to preserve fruits and vegetables but also fish, meat and dairy products and baked foods [1]. Although essential oils have been shown to be a promising alternative to chemical preservatives, they present concerns when added in foodstuff, due to their low water solubility, high volatility and strong odor that make them difficult for food applications.

As EOs are volatile compounds, they can easily evaporate or decompose owing to the exposure to heat, pressure, light or oxygen during food preparation or processing. Many antimicrobials of plant origin cannot be used in their free form due to their chemical instability, poor dispersibility in food matrices or unacceptable flavor profiles.

To avoid these drawbacks, encapsulation technologies, based on emulsion systems, have been developed and used industrially [10]. A well-designed delivery system can improve the handling, ease of use, stability and potency of plant-based preservatives.

Emulsions are extensively used for this purpose in many major industries such as pharmaceutical, food and biotechnology because they can be produced in a simple and manageable way using food grade ingredients [11,12,13].

In this direction, there is a growing interest in the development of alternative preservation methods involving the EOs and emulsion with interesting features in the field of coating material manufacturing. In fact, EOs represent great potential as additives in active food packaging since they can be dispersed as small droplets in a polymeric matrix leading to the formation of emulsion systems [14]. These systems effectively support the use of EOs in food by stabilizing them. Usually, the dispersed phase of oil in water emulsions consists of oil droplets, and the aqueous phase may consist of hydrophilic polymers, such as polysaccharides or proteins, to give to the continuous phase more stability [15]. Appropriate food grade surfactants (polysorbates, lecithins, saponins) may be incorporated to held together the two immiscible phases and to impart the desired features to the system [16]. An EO emulsion can be used for the preservation of food in different ways, for example by mixing it directly with liquid food or by washing food surface with antimicrobial dispersions, as well as by infusing it into porous food matrices and coating it with a biopolymer layer incorporating EO emulsions [17].

Among polysaccharides, alginate, isolated from bacteria and brown algae, is recognized as a versatile, non-toxic, low-cost biopolymer with interesting rheological properties [18]. This anionic polymer, composed of mannuronic and guluronic acid, is covalently bound together in different sequences. Alginate is used in a wide range of applications due in part to its ability to form hydrogels, which makes it attractive in drug delivery [19,20]. It is also added as an ingredient in food products where it can act as thickener or stabilizer.

In recent applications, alginate-based nanoemulsions enriched with essential oil were used as edible coating helping to extend the shelf-life of coated fruits, as well as for the production of self-standing films to be used as edible packaging [21]. Currently, we demonstrated that alginate-based nanoformulations in the presence of lemongrass essential oil (LEO) and Tween 80 had suitable rheological characteristics for being applied as a coating on fresh-cut apples by the dipping method [22].

Emulsion characteristics such as average droplet size and surface charge influence the transport of EOs to the cell membrane, as well as their interaction with the multiple molecular sites on the microbial cell membrane.

However, some studies have shown contradictory results; in some cases, the use of a nanoemulsion seemed to lower the antimicrobial activity [23] compared to the non-encapsulated compounds, while other studies showed higher antimicrobial activity of EOs when using nanoemulsions [24].

Even if essential oils showed a potential in food applications, usually their use in food industries is limited, as mentioned before, due to their strong flavor and aroma, but also due to their costs and toxicity at high concentrations. In fact, due to the interaction with macronutrients in foods, higher concentrations also have to be used to achieve the same inhibition effect in vitro.

Further studies have shown the importance of encapsulating essential oils in systems such as nanoformulations. In fact, thyme EO incorporated into an alginate-based coating was effective in inhibiting the growth of *L. monocytogenes*, *S. typhimurium*, *S. aureus* and *E. coli*, being a safe preservative for fresh-cut apples [25]. Kim and colleagues [26] showed that carnauba wax coating with lemongrass essential oil nanoemulsions completely inhibited the growth of *S. typhimurium* and *E. coli* in grape berries. A low concentration of oregano oil (0.1%) in nanoemulsion systems was effective against the same foodborne pathogens on fresh lettuce as reported by Bhargava et al. [27].

The aim of the present study was to evaluate the antifungal activity of nanoformulations sharing appropriate amounts of alginate and lemongrass essential oil. The inhibitory activity was evaluated against *Penicillium expansum*, *Aspergillus niger* and *Rhizopus* spp., typical molds responsible for food spoilage. *Penicillium expansum* is known as a post-harvest fungus responsible for fruits and vegetables spoilage and is also able to synthesize patulin, a mycotoxin whose toxicity to humans and animals has been demonstrated [28,29]. *Aspergillus niger* is a ubiquitous filamentous fungus that is commonly encountered in contaminated cereals, fruits, forages, vegetables and dairy products [30]. Furthermore, *Rhizopus* spp. although used in the production of fermented soy and dairy products, causes spoilage of bread, cereals, dairy products, fruits and vegetables [31,32].

## 2. Materials and Methods

### 2.1. Materials

Food-grade sodium alginate was supplied from Farmalabor (Canosa Di Puglia, Italy), Tween 80 (polyoxyethylene (20) sorbitan monooleate) was purchased from Sigma Aldrich (St. Louis, MO, USA), lemongrass (*Cymbopogon nardus*) essential oil (100%) obtained through steam distillation was from Erbamea (Lama di San Giustino, Italy).

### 2.2. Preparation of Nanoformulations

Sodium alginate solutions at different concentrations (1.0, 2.0, 3.0% *w*/*w*) were prepared by dissolving alginate powder in a water bath at 70 °C through gentle stirring with a magnetic bar. Nanoformulations were prepared using sodium alginate dispersion as the continuous phase and lemongrass essential oil (LEO) at different concentrations (0.1, 0.3, 0.7, 1.0, 1.5, 2.0% *w*/*w*) as the dispersed phase. All the nanoformulations were stabilized by Tween 80 (1% *w*/*w*). Coarse emulsions were prepared by mixing the aqueous phase with LEO and Tween 80 using a laboratory mixer, T25 digital Ultra-Turrax (IKA, Staufen, Germany), working at 24,000 rpm for 4 min. All the emulsions were then sonicated using an Ultrasonic Homogenizer (Model 300 VT -BioLogics, Manassas, VA, USA) for 1 min at 120 W with 50% pulsed frequency to reduce particle size. For all the preparations ultrapure water was used.

### 2.3. Nanoformulations Characterization

Rheological measurements of the obtained samples were carried out by means of a rotational rheometer, Haake MARS III (Thermo Scientific, Karlsruhe, Germany), 5 days after their preparation using a 60-mm diameter parallel plate geometry (PP60). The temperature was controlled by a Peltier system in combination with a water bath system (Phoenix II, Thermo Scientific, Karlsruhe, Germany). The samples (2.9 mL) were carefully poured onto the surface of the lower plate and the upper plate was lowered to 1 mm gap distance. Before testing, samples were left equilibrating for 5 min to allow for mechanical and temperature equilibration. Flow curves were made in control rate mode (CR) by varying the shear rate (0.1–1000 s^−1^) at 25 °C. The average size of the dispersed phase of nanoformulations used for antifungal activity was determined through dynamic light scattering (DLS) using a Malvern UK Zetasizer-Nano ZS 90 instrument (Malvern Instrument Ltd., Worcestershire, UK) operating with a 4 mW He-Ne laser (633 nm). The average diameter was measured at fixed detector angle of 90 by cumulant analysis of the autocorrelation function using software provided by the manufacturer. Before analysis samples were diluted 1:10 with ultra-pure water to avoid multiple scattering effects.

### 2.4. Antifungal Activity

#### 2.4.1. Microorganisms

Three fungal strains, *Penicillium expansum*, *Aspergillus niger*, and *Rhizopus* spp. were used in the study. The moulds were obtained from the Institute of Food Sciences of the National Research Council of Italy (Avellino) and were maintained on Potato Dextrose Agar (PDA) (Oxoid, Milan, Italy) at 25 °C.

#### 2.4.2. Antifungal Activity of Nanoformulations

The antifungal effect of nanoformulations at different concentrations of essential oil (0.1, 0.3, 0.7, 1.0, 1.5, 2.0%) was examined by agar spot assay. Considering the results of the rheological characterization, the nanoformulations were prepared with a fixed alginate concentration of 1%. Briefly, fungal spores obtained from 10-day-old cultures of the molds were used to inoculate in triplicate 20 mL of soft PDA medium poured into sterilized Petri dishes at a concentration of approximately 10^4^–10^5^ spore/mL. After solidification of the medium, 40 μL of the nanoformulations at different concentrations of LEO, were spotted onto agar plates. Alginate and Tween 80 (B2) and essential oil in sterile distilled water (A) were used as controls. The inoculated plates were incubated at 25 °C for ten days for fungal recovery. Inhibitory activity was determined by the presence or absence of fungal growth. All experiments were performed in duplicate.

## 3. Results and Discussion

### 3.1. Characterization of Alginate-LEO Based Nanoformulations

**Figure 1:**
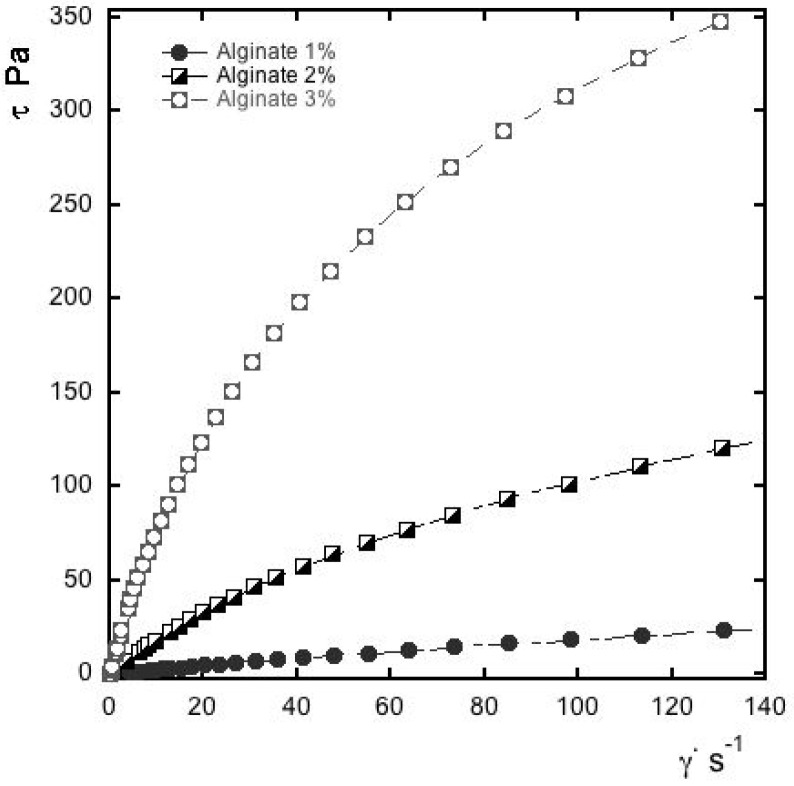
Flow curve of alginate at different concentrations of polymer (1, 2 and 3% *w*/*w*) homogenized and sonicated after the addition of Tween 80 (1% *w*/*w*).

Preliminary trials were made on dispersion in which the aqueous phase was at a different concentration of alginate (1, 2 and 3% *w*/*w*). Figure 1 depicts the flow curves of the dispersed systems (without essential oil), subjected to homogenization and sonication after the addition of the surfactant. The pseudoplastic behavior typical of alginate is more visible as the concentration of polysaccharide increases.

Considering this, the concentration of 1% *w*/*w* alginate was chosen as a continuous phase in view of a future application of the essential oil-enriched dispersions. Indeed, the rheological characteristics of nanoformulations have a certain importance in the current food application, and the flow curves of emulsified system can reflect their stability [33,34]. Furthermore, these features affect the appearance, texture and mouthfeel of samples, thus affecting their overall workability and quality attributes [35].

Then, 1% alginate nanoformulations, enriched with the different concentrations of essential oil and stabilized with Tween 80 (1% *w*/*w*), were characterized. In detail, the rotational tests here reported are those performed on nanoformulations with LEO concentrations effective for a reasonable mold inhibition (i.e., 1, 1.5 and 2%, see antifungal results) characterized by values of particle size of 434 nm, 509 nm and 608 nm for 1, 1.5 and 2% of essential oil, respectively.

**Figure 2:**
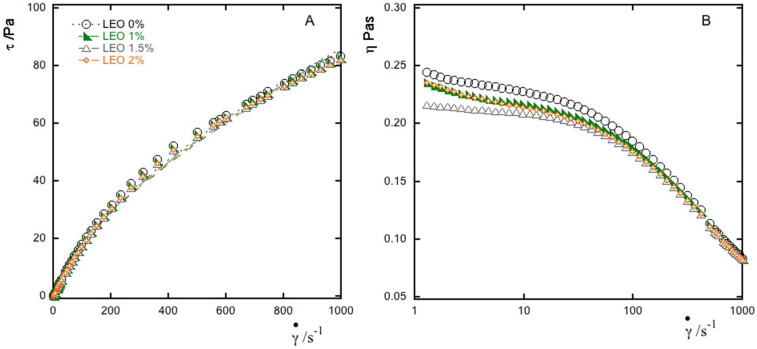
Flow curve (**A**) and apparent viscosity curve; (**B**) of alginate nanoformulations at different concentration of LEO (0%-empty circle, 1%-green triangle, 1.5%-empty triangle, 2%-orange cross) stabilized by Tween 80 (1% *w*/*w*).

All samples showed non-Newtonian pseudo plastic behavior, as for the alginate suspensions. In Figure 2A, flow curves of the dispersions at different LEO concentrations showed the shear stress as function of shear rate, while Figure 2B reported the viscosity curves where it is easy to see how viscosity values decrease with the shear rate increase, enhancing the shear thinning behavior [22].

Rheological flow properties were adjusted according to the Ostwald-de Waele model, widely used to describe rheological behaviors of food emulsions, according to Equation (1) [36,37].

(1) $$\tau =k{\dot{\gamma }}^{n}$$

where *τ* is the shear stress (Pa), $\dot{\gamma }$ is the shear rate (s^−1^), *k* is known as the consistency index (Pa × s^*n*^) corresponding to the fluid consistency and *n* is known as the flow behavior index (dimensionless number). The constant *n* determines the behavior of a fluid; when *n* is 1 the fluid behaves as a Newtonian fluid and the consistency index will coincide with the viscosity value, for *n* values < 1 the fluid is considered a shear thinning pseudo plastic fluid; for *n* values > 1 the fluid rheogram will show the behavior of a shear thickening or dilatant fluid.

Table 1 summarizes the results obtained from the flow curve fittings, where the power law constants *n* and *k* aid in understanding the rheological behavior of the emulsions analyzed.

The flow indices are all less than 1 regardless of the LEO concentration, indicating that the behavior deviates from the Newtonian one. The values of consistency index reflect the viscosity values of the samples studied, and the results obtained showed that the presence of essential oil slightly influences the *k* values, which decrease as the LEO content increases. A fundamental role is played by the continuous phase on the nanoformulations characteristics and regulates their behavior during processing [38]. The results indicate that the rheology of the emulsified systems was dominated by the anionic polymer while the presence of oil did not really influence the behavior also due to the relatively low level of oil droplets (<2% *w*/*w*) [39]. Wu et al. [40] observed that, when incorporating essential oil into a polymeric matrix, a pseudo plastic behavior is visible, influenced by the addition of EO, which caused an increase in *n* value. Moreover, other authors observed an increase in the viscosity value, usually related to the use of a much higher oil content [41]. The wide range of shear rates used allows us to see that the alginate-based nanoformulations produced are stable even under different stress conditions, which are supposed to mimic some processes that occurs in food production, for example stirring or mixing in a vessel, pouring from a bottle or mastication of a food [42].

### 3.2. Antifungal Activity

Based on the previously obtained viscosity values, only nanoformulations with 1% alginate as a continuous phase were tested, while the amount of essential oil varied from 0 to 2%. Main components of the *Cymbopogon nardus* essential oil are monoterpenes, hydrocarbons and aldehydes. As reported by other authors [43,44], citronellal, geraniol and citronellol are present at higher concentrations, and variation in their amount might arise from environmental and genetic differences. In particular, citronellal as major component, plays a key role in the antifungal activity, together with the synergic activity of the minor constituents [45].

The effectiveness of the nanoformulations with different concentrations of lemon grass essential oil was tested against *P. expansum*, *A. niger* and *Rhizopus* spp. The choice of these molds was related to the fact that the developed nanoformulations can be used for postharvest applications on fresh products and, as mentioned before, the three fungi can cause fruit rot or spoilage of different food products.

The ability of the nanoformulations to inhibit fungal growth was monitored for 10 days. Table 2 shows the results after 3 and 10 days of contact between the nanoformulations and the fungi. The inhibition depended on the percentage of essential oil dispersed in the nanoformualtions. After three days of incubation, all fungi were inhibited by nanoformulations with LEO concentrations above 1%. Concentrations below 0.7% did not inhibit any molds (see Table 2).

**Figure 3:**
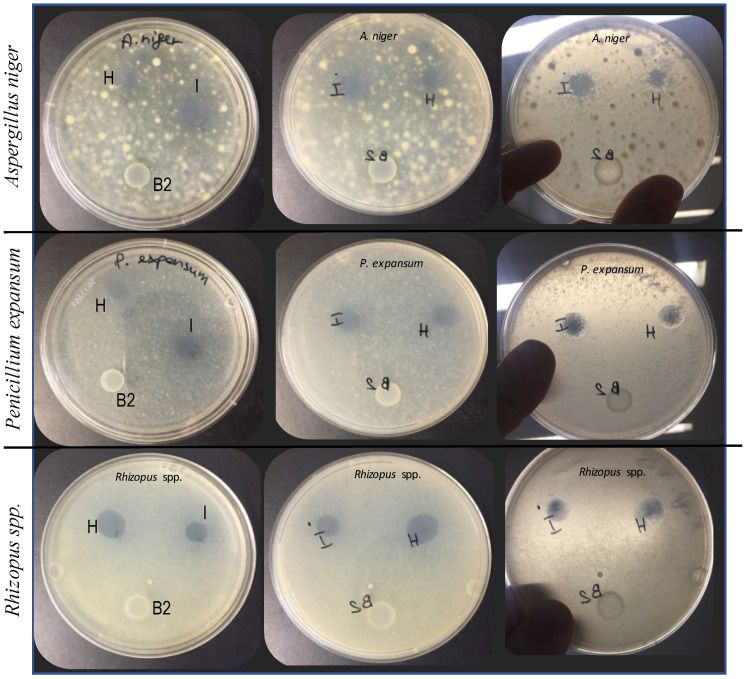
Mycelial growth inhibition of nanoformulations of lemon grass essential oil at different concentrations against *Aspergillus niger*, *Penicillium expansum* and *Rhizopus* spp. after 3 days of incubation at 25 °C. B2 = control spot containing Alg/tween; H = spot of nanoformulations with 1.5% LEO, I = spot of nanoformulations with 2% LEO. The first column shows the front side of the Petri dish, the second and third columns show the back side of the Petri dish.

Already after three days of incubation, when the grow of molds began to be visible, it was possible to see a clear absence of growth where the nanoformulations with essential oil were spotted (Figure 3).

Figure 3 shows, for all the fungi tested, the absence of fungal growth on 1.5% (H) and 2% (I) essential oil spot, while on the control spot consisting only of the alginate/tween system the molds grew (B2).

Interestingly, as shown in Table 2, the inhibiting activity of the nanoformulations was still active after 10 days of incubation, especially against *Penicillium expansum* and *Rhyzopus* spp. for which the lowest inhibiting concentration remained at 1%. On the other hand, after 10 days of incubation, the lowest percentage of essential oil able to inhibit *A. niger* was 1.5%.

**Figure 4:**
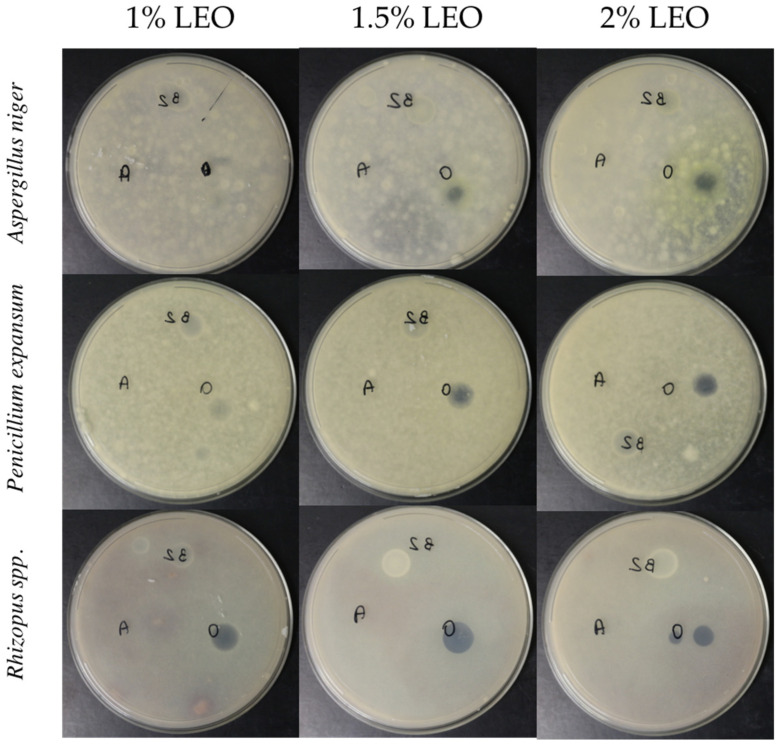
Mycelial growth inhibition of nanoformulations at different concentrations of lemon grass essential oil against *Aspergillus niger, Penicillium expansum* and *Rhizopus* spp. after 9 days of incubation at 25 °C. B2 = control spot containing alginate/Tween 80; A = essential oil in water, O = essential oil nanoformulation.

In order to evaluate the effectiveness of the nanoformulations systems, a spot with pure essential oil at the same concentrations used in the nanoformulations was tested against the molds. As shown in Figure 4, the lemongrass essential oil dispersed in the nanoformulations was able to inhibit the growth of the three molds tested until 10 days’ incubation, while the same LEO concentration dispersed in distilled water did not produce the same effect (A).

In particular, an inhibitory effect against *Rhyzopus* spp. was already obtained with the 1% essential oil nanoformulation, while for *A. niger* and *P. expansum* it was necessary to reach at least 1.5% essential oil concentration to achieve an inhibitory effect. This study showed that alginate assisted lemongrass essential oil dispersions in their anti-fungal activity. Indeed, it was observed that neither the system with only alginate and surfactant (B2) nor the essential oil dispersed in water (A) exhibited any inhibitory effect (Figure 4). Thus, the same amount of essential oil causing growth inhibition of different fungi does not work if simply mixed in water due to rapid volatilization.

**Figure 5:**
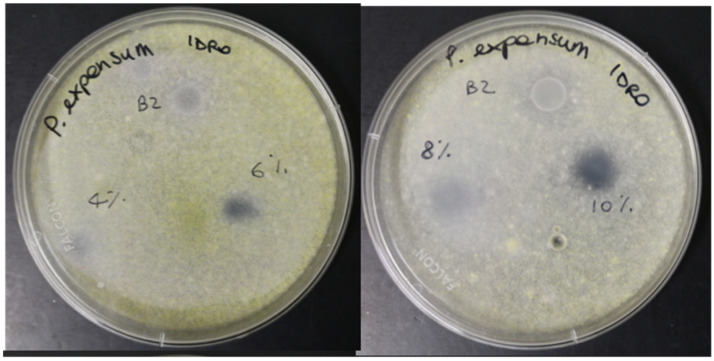
Mycelial growth inhibition of lemon grass essential oil dispersed in water at different concentrations (4, 6, 8, 10%) against *Penicillium expansum* after 9 days of incubation at 25 °C. B2 = control spot containing alginate/Tween 80.

Furthermore, we observed that to obtain the same inhibitory effect of the alginate assisted lemongrass essential oil dispersion, the EO dissolved in water should be used in concentrations higher than 6%. Figure 5 shows, in fact, that the mycelial growth of *Penicillium expansum* after 10 days of incubation was inhibited when the essential oil concentration in water was at least 6%.

According to Donsì et al. [17], small particles with hydrophilic surface of the nanoformulations are able to pass through the cell membrane. The fusion of the emulsifier droplets with the phospholipid bilayer of the microorganisms facilitates their accessibility through the cell membrane surface, allowing the rupture of the latter and leading to cell death.

Other studies have also shown that encapsulation of EOs in colloidal systems such as nanoformulations leads to higher antimicrobial activity compared to the use of essential oil alone [46,47].

Thus, the possibility of developing nanoformulations of essential oils may not only significantly improve the antifungal activity but also require smaller amounts of essential oil for the development of an effective formulation. However, several factors can influence the antimicrobial efficiency of essential oils nanoformulations as also reported by Bedoya et al. [48], who highlighted that the formulation, the average particle size and the surface charge influence the antimicrobial activity of the essential oil.

## 4. Conclusions

The restrictions on the use of some synthetic food additives, imposed by food industry and regulatory agencies, have led to renewed interest in searching for alternative antimicrobial compounds for use in food, such as essential oils. However, the biological activity of EOs could be lost by volatilization of active components or other degradation reactions. Thus, the incorporation of essential oil in emulsion based nanoformulation systems, to protect and use it in lower quantities, represents a valid prospect to overcome the disadvantages that limit the commercial application of EOs.

Nanoformulations are colloidal dispersions characterized by a continuous phase in which the dispersed phase, such as the lipophilic active ingredient, is stabilized by a surfactant and reduced to small droplets to increase its solubility, stability and possibly enhance its biological activity.

Based on the already proven effectiveness of the alginate-based nanoformulation enriched with essential oil, which can be used as an edible film and edible coating, extending the shelf life of fresh-cut fruits, this study also underlines the importance of emulsified systems on the antifungal properties of active compounds, such as EOs.

To this end, the antifungal activity of nanoformulations at different concentrations of lemongrass essential oil with 1% alginate as continuous phase, stabilized by Tween 80 (1% *w*/*w*) was investigated.

With regard to the rheological properties of the samples, these were not influenced by the concentration of active compound, thus allowing the systems proposed here to be used in various applications and also indicating their stability at different shear stress conditions.

In this study, we demonstrated that emulsion based nanoformulations sharing the following composition 1%alginate/1.5%LEO/1%Tween 80 favors the inhibitory action of lemongrass (*Cymbopogon nardus*) essential oil against *Rhyzopus* spp., *Penicillium expansum* and *Aspergillus niger*, for at least 10 days of incubation. In addition to the positive preliminary in vitro results, future research is mainly needed to evaluate the in vivo antifungal activity of the proposed systems.

## Figures and Tables

**Table 1:** Consistency index (*k*) and flow index (*n*) of alginate/lemongrass essential oil (LEO) nanoformulations obtained from the fitting with power law.

	LEO 0%	LEO 1%	LEO 1.5%	LEO 2%
*k*	0.96 ± 0.06	0.90 ± 0.06	0.86 ± 0.06	0.88 ± 0.06
*n*	0.65 ± 0.01	0.66 ± 0.01	0.66 ± 0.01	0.66 ± 0.01
*r* ^2^	0.997	0.997	0.997	0.997

**Table 2:** Inhibition of mold growth after 3 days and 10 days of incubation.

	After 3 Days of Incubation						After 10 Days of Incubation					
	LEO (%)						LEO (%)					
	0.1	0.3	0.7	1	1.5	2	0.1	0.3	0.7	1	1.5	2
*Aspergillus niger*	−	−	−	+	+	+	−	−	−	−	+	+
*Penicillium expansum*	−	−	−	+	+	+	−	−	−	+/−	+	+
*Rhizopus* spp.	−	−	−	+	+	+	−	−	−	+	+	+

(−) no mold inhibition; (+) mold inhibition; (+/−) moderate mold inhibition.

## Data Availability

Not applicable.

## References

[1] Sharma S., Barkauskaite S., Jaiswal A.K., Jaiswal S. (2020). Essential oils as additives in active food packaging. Food Chem..

[2] Chouhan S., Sharma K., Guleria S. (2017). Antimicrobial Activity of Some Essential Oils—Present Status and Future Perspectives. Medicines.

[3] Sandner G., Heckmann M., Weghuber J. (2020). Immunomodulatory activities of selected essential oils. Biomolecules.

[4] De Lavor É.M., Fernandes A.W.C., de Andrade Teles R.B., Leal A.E.B.P., de Oliveira Júnior R.G., Gama e Silva M., De Oliveira A.P., Silva J.C., de Moura Fontes Araújo M.T., Coutinho H.D.M. (2018). Essential oils and their major compounds in the treatment of chronic inflammation: A review of antioxidant potential in preclinical studies and molecular mechanisms. Oxidative Med. Cell. Longev..

[5] López P., Sánchez C., Batlle R., Nerín C. (2007). Development of flexible antimicrobial films using essential oils as active agents. J. Agric. Food Chem..

[6] Spisni E., Petrocelli G., Imbesi V., Spigarelli R., Azzinnari D., Donati Sarti M., Campieri M., Valerii M.C. (2020). Antioxidant, Anti-Inflammatory, and Microbial-Modulating Activities of Essential Oils: Implications in Colonic Pathophysiology. Int. J. Mol. Sci..

[7] Nazzaro F., Fratianni F., Coppola R., Feo V.D. (2017). Essential oils and antifungal activity. Pharmaceuticals.

[8] Tiwari B.K., Valdramidis V.P., O’Donnell C.P., Muthukumarappan K., Bourke P., Cullen P. (2009). Application of natural antimicrobials for food preservation. J. Agric. Food Chem..

[9] Mutlu-Ingok A., Devecioglu D., Dikmetas D.N., Karbancioglu-Guler F., Capanoglu E. (2020). Antibacterial, antifungal, antimycotoxigenic, and antioxidant activities of essential oils: An updated review. Molecules.

[10] Doost A.S., Nasrabadi M.N., Kassozi V., Nakisozi H., Van der Meeren P. (2020). Recent advances in food colloidal delivery systems for essential oils and their main components. Trends Food Sci. Technol..

[11] Cuomo F., Perugini L., Marconi E., Messia M.C., Lopez F. (2019). Enhanced curcumin bioavailability through nonionic surfactant/caseinate mixed nanoemulsions. J. Food Sci..

[12] Mosca M., Diantom A., Lopez F., Ambrosone L., Ceglie A. (2013). Impact of antioxidants dispersions on the stability and oxidation of water-in-olive-oil emulsions. Eur. Food Res. Technol..

[13] Cuomo F., Cinelli G., Chirascu C., Marconi E., Lopez F. (2020). Antioxidant effect of vitamins in olive oil emulsion. Colloids Interfaces.

[14] Cuomo F., Iacovino S., Messia M.C., Sacco P., Lopez F. (2020). Protective action of lemongrass essential oil on mucilage from chia (*Salvia hispanica*) seeds. Food Hydrocoll..

[15] McClements D.J., Rao J. (2011). Food-grade nanoemulsions: Formulation, fabrication, properties, performance, biological fate, and potential toxicity. Crit. Rev. Food Sci. Nutr..

[16] Doost A.S., Van Camp J., Dewettinck K., Van der Meeren P. (2019). Production of thymol nanoemulsions stabilized using Quillaja Saponin as a biosurfactant: Antioxidant activity enhancement. Food Chem..

[17] Donsi F., Ferrari G. (2016). Essential oil nanoemulsions as antimicrobial agents in food. J. Biotechnol..

[18] Cofelice M., Lopez F., Cuomo F. (2018). Rheological Properties of Alginate–Essential Oil Nanodispersions Colloids Interfaces. Colloids Interfaces.

[19] Farrés I.F., Norton I. (2014). Formation kinetics and rheology of alginate fluid gels produced by in-situ calcium release. Food Hydrocoll..

[20] Cong Z., Shi Y., Wang Y., Wang Y., Chen N., Xue H. (2018). A novel controlled drug delivery system based on alginate hydrogel/chitosan micelle composites. Int. J. Biol. Macromol..

[21] Cofelice M., Cuomo F., Chiralt A. (2019). Alginate Films Encapsulating Lemongrass Essential Oil as Affected by Spray Calcium Application. Colloids Interfaces.

[22] Cofelice M., Lopez F., Cuomo F. (2019). Quality Control of Fresh-Cut Apples after Coating Application. Foods.

[23] Shah B., Davidson P.M., Zhong Q. (2013). Nanodispersed eugenol has improved antimicrobial activity against *Escherichia coli* O157: H7 and *Listeria monocytogenes* in bovine milk. Int. J. Food Microbiol..

[24] Xue J., Davidson P.M., Zhong Q. (2015). Antimicrobial activity of thyme oil co-nanoemulsified with sodium caseinate and lecithin. Int. J. Food Microbiol..

[25] Hu W., Feng K., Xiu Z., Jiang A., Lao Y. (2019). Efficacy of thyme oil-alginate-based coating in reducing foodborne pathogens on fresh-cut apples. Int. J. Food Sci. Technol..

[26] Kim I.-H., Oh Y.A., Lee H., Song K.B., Min S.C. (2014). Grape berry coatings of lemongrass oil-incorporating nanoemulsion. LWT Food Sci. Technol..

[27] Bhargava K., Conti D.S., da Rocha S.R., Zhang Y. (2015). Application of an oregano oil nanoemulsion to the control of foodborne bacteria on fresh lettuce. Food Microbiol..

[28] Morales H., Sanchis V., Usall J., Ramos A.J., Marín S. (2008). Effect of biocontrol agents *Candida sake* and *Pantoea agglomerans* on *Penicillium expansum* growth and patulin accumulation in apples. Int. J. Food Microbiol..

[29] Sorrentino E., Reale A., Tremonte P., Maiuro L., Succi M., Tipaldi L., Di Renzo T., Pannella G., Coppola R. (2013). *Lactobacillus plantarum* 29 Inhibits *Penicillium* spp. Involved in the Spoilage of Black Truffles (*Tuber aestivum*). J. Food Sci..

[30] Sebti I., Martial-Gros A., Carnet-Pantiez A., Grelier S., Coma V. (2005). Chitosan polymer as bioactive coating and film against Aspergillus niger contamination. J. Food Sci..

[31] Saleh I., Al-Thani R. (2019). Fungal food spoilage of supermarkets’ displayed fruits. Vet. World.

[32] Snyder A.B., Worobo R.W. (2018). Risk mitigation for immunocompromised consumers of mucormycete spoiled and fermented foods: Germane guidance and remaining needs. Microorganisms.

[33] Floury J., Desrumaux A., Lardières J. (2000). Effect of high-pressure homogenization on droplet size distributions and rheological properties of model oil-in-water emulsions. Innov. Food Sci. Emerg. Technol..

[34] Kawada H., Kume T., Matsunaga T., Iwai H., Sano T., Shibayama M. (2010). Structure and rheology of a self-standing nanoemulsion. Langmuir.

[35] Zhu Y., Gao H., Liu W., Zou L., McClements D.J. (2020). A review of the rheological properties of dilute and concentrated food emulsions. J. Texture Stud..

[36] Alarcón-Moyano J., Matiacevich S. (2019). Active emulsions based on alginate and lemongrass/citral essential oils: Effect of encapsulating agents on physical and antimicrobial properties. Int. J. Food Prop..

[37] Ma J., Lin Y., Chen X., Zhao B., Zhang J. (2014). Flow behavior, thixotropy and dynamical viscoelasticity of sodium alginate aqueous solutions. Food Hydrocoll..

[38] Rodríguez-Abreu C., Lazzari M. (2008). Emulsions with structured continuous phases. Curr. Opin. Colloid Interface Sci..

[39] Salvia-Trujillo L., Decker E.A., McClements D.J. (2016). Influence of an anionic polysaccharide on the physical and oxidative stability of omega-3 nanoemulsions: Antioxidant effects of alginate. Food Hydrocoll..

[40] Wu C., Wang L., Hu Y., Chen S., Liu D., Ye X. (2016). Edible coating from citrus essential oil-loaded nanoemulsions: *Physicochemical characterization* and preservation performance. RSC Adv..

[41] Barradas T.N., de Campos V.E.B., Senna J.P., Coutinho C.d.S.C., Tebaldi B.S., e Silva K.G.d.H., Mansur C.R.E. (2015). Development and characterization of promising o/w nanoemulsions containing sweet fennel essential oil and non-ionic sufactants. Colloids Surf. A Physicochem. Eng. Asp..

[42] Vanapalli S.A., Coupland J.N., Friberg S., Larsson K. (2004). Orthokinetic stability of food emulsions. Food Emuls..

[43] de Billerbeck V.G., Roques C.G., Bessière J.-M., Fonvieille J.-L., Dargent R. (2001). Effects of *Cymbopogon nardus* (L.) W. Watson essential oil on the growth and morphogenesis of Aspergillus niger. Can. J. Microbiol..

[44] Mahalwal V.S., Ali M. (2003). Volatile constituents of *Cymbopogon nardus* (Linn.) Rendle. Flavour Fragr. J..

[45] Aguiar R.W.d.S., Ootani M.A., Ascencio S.D., Ferreira T.P., Santos M.M.d., Santos G.R.d. (2014). Fumigant antifungal activity of *Corymbia citriodora* and *Cymbopogon nardus* essential oils and citronellal against three fungal species. Sci. World J..

[46] Anwer M.K., Jamil S., Ibnouf E.O., Shakeel F. (2014). Enhanced antibacterial effects of clove essential oil by nanoemulsion. J. Oleo Sci..

[47] Pongsumpun P., Iwamoto S., Siripatrawan U. (2020). Response surface methodology for optimization of cinnamon essential oil nanoemulsion with improved stability and antifungal activity. Ultrason. Sonochem..

[48] Bedoya-Serna C.M., Dacanal G.C., Fernandes A.M., Pinho S.C. (2018). Antifungal activity of nanoemulsions encapsulating oregano (*Origanum vulgare*) essential oil: In vitro study and application in Minas Padrão cheese. Braz. J. Microbiol..

